# Regulation and Cytoprotective Role of Hexokinase III

**DOI:** 10.1371/journal.pone.0013823

**Published:** 2010-11-03

**Authors:** Eugene Wyatt, Rongxue Wu, Wael Rabeh, Hee-Won Park, Mohsen Ghanefar, Hossein Ardehali

**Affiliations:** 1 Division of Cardiology, Department of Medicine, Northwestern University Feinberg School of Medicine, Chicago, Illinois, United States of America; 2 Structural Genomics Consortium, University of Toronto, Toronto, Canada; 3 Department of Pharmacology, University of Toronto, Toronto, Canada; Universität Heidelberg, Germany

## Abstract

**Background:**

Hexokinases (HKs) catalyze the first step in glucose metabolism. Of the three mammalian 100-kDa HK isoforms, HKI and II can bind to mitochondria and protect against cell death. HKIII does not bind mitochondria, and little is known about its regulation or cytoprotective effects. We studied the regulation of HKIII at the transcriptional and protein levels and investigated its role in cellular protection.

**Methodology/Principal Findings:**

We show that like HKII, HKIII expression is regulated by hypoxia, but other factors that regulate HKII expression have no effect on HKIII levels. This transcriptional regulation is partially dependent on hypoxia-inducible factor (HIF) signaling. We also demonstrate regulation at the protein level, as mutations in putative N-terminal substrate binding residues altered C-terminal catalytic activity, suggesting that HKIII activity is governed, in part, by interactions between these two domains. Overexpression of HKIII reduced oxidant-induced cell death, increased ATP levels, decreased the production of reactive oxygen species (ROS), and preserved mitochondrial membrane potential. HKIII overexpression was also associated with higher levels of transcription factors that regulate mitochondrial biogenesis, and greater total mitochondrial DNA content. Attempts to target HKIII to the mitochondria by replacing its N-terminal 32-amino-acid sequence with the mitochondrial-targeting sequence of HKII led to protein aggregation, suggesting that this region is necessary to maintain proper protein folding and solubility.

**Conclusions/Significance:**

These results suggest that HKIII is regulated by hypoxia and there are functional interactions between its two halves. Furthermore, HKIII exerts protective effects against oxidative stress, perhaps by increasing ATP levels, reducing oxidant-induced ROS production, preserving mitochondrial membrane potential, and increasing mitochondrial biogenesis.

## Introduction

Hexokinases (HKs or ATP:D-hexose 6-phosphotransferase [EC 2.7.1.1] in IUB nomenclature) transfer a phosphoryl moiety from ATP to the 6-hydroxyl of glucose to produce glucose-6-phosphate (G6P). The glucose phosphorylation reaction carried out by HKs maintains the concentration gradient necessary for facilitative glucose transporters (GLUTs) to move glucose into the cell [1], [2], [3], [4]. There are 4 mammalian hexokinase isozymes: HKI, II, III, and IV; hexokinase IV is also known as glucokinase (GK). The molecular weights of HKI, II, and III are approximately 100-kDa, whereas HKIV has a molecular weight of 50-kDa. The three 100-kDa HK isozymes have extensive sequence homology and a high affinity for glucose (K_m_<1 mM), and are inhibited by the reaction product G6P; however, differences in tissue expression, cellular distribution, and regulation suggest that each isozyme has a distinct metabolic role [5], [6], [7]. HKI is ubiquitously expressed, while HKII is primarily expressed in insulin sensitive tissue such as skeletal and cardiac muscle. HKIII expression is relatively low in most tissue, with the highest levels reported in lung, kidney, and liver [7], [8], [9]. HKIV has a low affinity for glucose, is not inhibited by G6P, and is expressed mainly in pancreatic B cells and the liver, where it is believed to function as a ‘glucose sensor’ [8].

The 100-kDa HK isozymes are each composed of two 50-kDa domains and may have evolved from the genetic duplication and fusion of an ancestral 50-kDa HK. Early descriptions of these enzymes proposed that one of the 50-kDa subunits retained catalytic activity, and the other became the regulatory domain [7], [8]. Only the C-terminal domain of HKI is catalytically active, but G6P binds to both domains [10], [11], [12], [13], [14], [15], [16]. In HKII, both domains are catalytically active and sensitive to G6P, and glucose binding to the N-terminal domain decreases the concentration of G6P required to regulate the activity of the C-terminal domain, suggesting that the two halves functionally interact [17], [18]. Like HKI, the catalytic activity of HKIII is restricted to its C-terminal half [17], [19], [20].

HKI and HKII also contain a conserved 21-amino-acid sequence in the N-terminal domain that is predicted to form a hydrophobic α-helix and enables these proteins to bind to mitochondria [21], [22]. HKIII does not contain the conserved mitochondrial-binding sequence and is cytoplasmic, although some investigations report perinuclear binding [23]. One consequence of HKI and HKII binding to the mitochondria is the prevention of cell death, possibly through interactions with the voltage-dependent anion channel and inhibition of the mitochondrial permeability transition pore, which is a pro-death channel [24], [25]. Overexpression of HKI and HKII in tissue culture protects against cell death [26], and both the mitochondrial-binding and glucose-phosphorylation activities of these proteins are required for their protective effects [27].

The regulation of HKIII and its potential role in cytoprotection are not well characterized. In this report, we studied the regulation of HKIII at the transcriptional and protein levels as well as its role in cellular protection. We show that of the various factors that regulate HKII, only hypoxia influences HKIII levels through a HIF-dependent pathway. Mutations in the predicted N-terminal glucose and G6P binding sites of HKIII decreased activity in the C-terminus, which suggests that the two halves of HKIII functionally interact. HKIII also protects against cell death, and its overexpression increases ATP levels, decreases the oxidant-induced production of reactive oxygen species (ROS), attenuates the oxidant-induced reduction in mitochondrial membrane potential, and increases mitochondrial biogenesis. To better characterize the importance of mitochondrial binding on the protective effects of HKs, we attempted to target HKIII to the mitochondria by replacing the N-terminal 32-amino-acid region of HKIII with the mitochondrial binding domain of HKII. However, the resultant chimeric protein aggregated, suggesting that unlike HKI and HKII, the N-terminal region of HKIII may play a role in tertiary structure formation and in maintaining protein solubility.

## Methods

### Cell culture

N1S1 cells are descended from the Novikoff rat hepatoma cell line and were a gift from Dr. Andrew Larson (Northwestern); the cells were maintained as previously described [28]. HEK293 (HEK) cells were obtained from the American Type Culture Collection (ATCC) and maintained in MEM (Cellgro, Mediatech Inc.) supplemented with 10% fetal bovine serum (Cellgro, Mediatech, Inc.); sodium pyruvate; and penn/strep (Gibco, Invitrogen Corporation).

### Cell treatments

For the hypoxia studies, cells were incubated at 1.5% O_2_ for 24 hours. To stabilize HIF isoforms under normoxic conditions, cells were incubated with 100 or 300 µM of the prolyl hydroxylase inhibitor Dimethyloxalyl glycine (DMOG) (Roche) for 24 h before isolation of mRNA and protein. For evaluation of cAMP or AMP-activated kinase (AMPK) activation, N1S1 cells were incubated for 24 hours in serum free media with 5 µM forskolin or 1 mM aminoimidazole carboxamide ribonucleotide (AICAR), respectively (Sigma).

### RNA isolation and Western blotting

Total RNA from cells was extracted with RNA STAT (Tel-test Inc) and reverse transcribed with the TaqMan preamplification system (Applied Biosystems). Real time polymerase chain reaction (PCR) was performed using SYBR Green and assayed on the ABI 7500 Detector (Applied Biosystems). Primer sequences for all targets are listed in Supplemental Table S1. All target mRNA levels were normalized to an interal housekeeping control (18S or β2M). For Western blot, cells were lysed and protein samples were run on an SDS PAGE gel. Western blots were performed using commercially available antibodys for HKIII and actin (Abcam, and Santa Cruz respectively).

### HKIII and HIF constructs

A plasmid containing HKIII cDNA was kindly provided by Dr. Graeme Bell (University of Chicago). 5′ EcoR1 and 3′ BamH1 sites were engineered into the sequence around the region of interest, then DNA was amplified via PCR and gel purified. The construct was cloned into the TOPO vector (Invitrogen), and the DNA sequence was confirmed. Using this construct as a template, we introduced ATG codons into PCR primers that initiated protein translation at amino-acid positions 9, 17, 25, and 33, thereby generating truncated versions of the HKIII protein lacking the first 8, 16, 24, and 32 residues of the native sequence, respectively. To generate a chimeric HKIII protein containing the HKII mitochondrial-targeting sequence, the construct lacking the first 32 amino acids was used as a template with primers encoding the 21-amino-acid N-terminal region of HKII. After all DNA sequences were confirmed, the HKIII fragments were excised via restriction digestion and subcloned into the pEGFP-N3 vector (Clontech). Constitutively active HIF-1α and HIF-2α constructs have double proline-to-alanine point mutations preventing hydroxylation and subsequent degradation [29]. These constructs were generously donated by Dr. Mircea Ivan (Indiana University).

### Viability assays

The HKIII-GFP fusion construct or the GFP vector was transfected into HEK using Lipofectamine2000 (Invitrogen). Expression was confirmed with fluorescent microscopy, and Western blot analyses of lysates from transfected cells as previously described [27]. Experiments were performed 36 to 48 hours after transfection, and cells were cultured overnight in basal MEM without supplements before each experiment. To induce death, cells were exposed to exogenous oxidative stress using H_2_O_2_ at the dose and time points indicated. Cell viability was assessed using propidium iodide exclusion and Annexin V conjugated to Alexa-350 (Invitrogen) according to the manufacturer's instructions. Results were quantified via flow cytometry using the BDFACS LSR flowcytometer and analyzed using the FlowJo software program.

### ATP assessment

ATP levels were determined using the Cell Titer Glo Assay from Promega, according to the manufacturer's recommendations. Luminescence was recorded with the SpectraMax Gemini (Molecular Devices), and measurements were normalized to the total number of viable cells in each sample, as determined by trypan blue exclusion with cell counts made on a hemocytometer.

### ROS measurements

ROS levels were measured using the MitoSOX fluorescent marker (Invitrogen), which selectively reacts with superoxide. Briefly, the MitoSox probe (1 µM) was added to cultured cells 10 min prior to the end of the indicated treatment condition. Cells were then washed with PBS and incubated for 5 min in MEM containing the Hoechst33342 nuclear marker (Invitrogen). The media was replaced with fresh MEM and ROS levels were evaluated via fluorescent microscopy. Images from 5 representative fields were captured for each individual sample, and fluorescent intensity in the 510/580 nm spectra was evaluated using the ImageJ software program (NIH), and normalized to cell number.

### Mitochondrial Membrane Potential

Mitochondrial membrane potential (*Δψ*_m_), was evaluated using the fluorescent marker tetramethylrhodamine ethyl ester TMRE (Sigma) as previously described [27]. Briefly, cells were loaded with TMRE 10 min prior to the start of the indicated treatment. Fluorescent microscopy images were taken to demonstrate relative changes in TMRE intensity. Results were quantified via flow cytometry as describe above.

### Mitochondrial DNA content

Mitochondrial biogenesis was assessed by determining the mitochondrial DNA copy number. Total DNA was isolated using a modification of the spin-column protocol from the DNeasy Blood and Tissue Kit (Qiagen), then the quantity of the mitochondrial encoded gene cytochrome c oxidase subunit 1 (CO1) and the quantity of the stable nuclear-encoded gene 18S ribosomal RNA (rRNA) were measured via PCR. Mitochondrial DNA copy number was expressed as the ratio of CO1 to 18SrRNA expression.

### Hexokinase activity

The QuikChange II Multi-site Directed Mutagenesis Kit (Stratagene) was used to make point mutations in HKIII substrate-binding residues. The full-length HKIII protein was used as the template. HKIII constructs were PCR-amplified from human genomic DNA and subcloned into pET28a (Novagen). Proteins were expressed in E. coli (BL21 DE3) and purified with Ni-NTA affinity chromatography and Superdex 75 (26/60) size-exclusion chromatography (pre-equilibrated with 10 mM Tris pH 7.5, 150 mM NaCl, 10 mM MgCl2, and 5 mM DTT). Proteins were concentrated using an Amicon Ultra centrifugal filter with purity >95% based on sodium dodecyl sulfate polyacrylamide gel electrophoresis (SDS-PAGE) analysis. HK activity was assessed as described previously [17] by coupling G6P production with NADP reduction in the presence of G6P dehydrogenase (G6PDH).

### Statistical analysis

Results are presented as mean±SEM. The Student's t-test was used to analyze data with unequal variance between each group. A p-value of less than 0.05 was considered significant.

## Results

### Hypoxia induces HKIII expression through HIF dependent signaling

**Figure 1:**
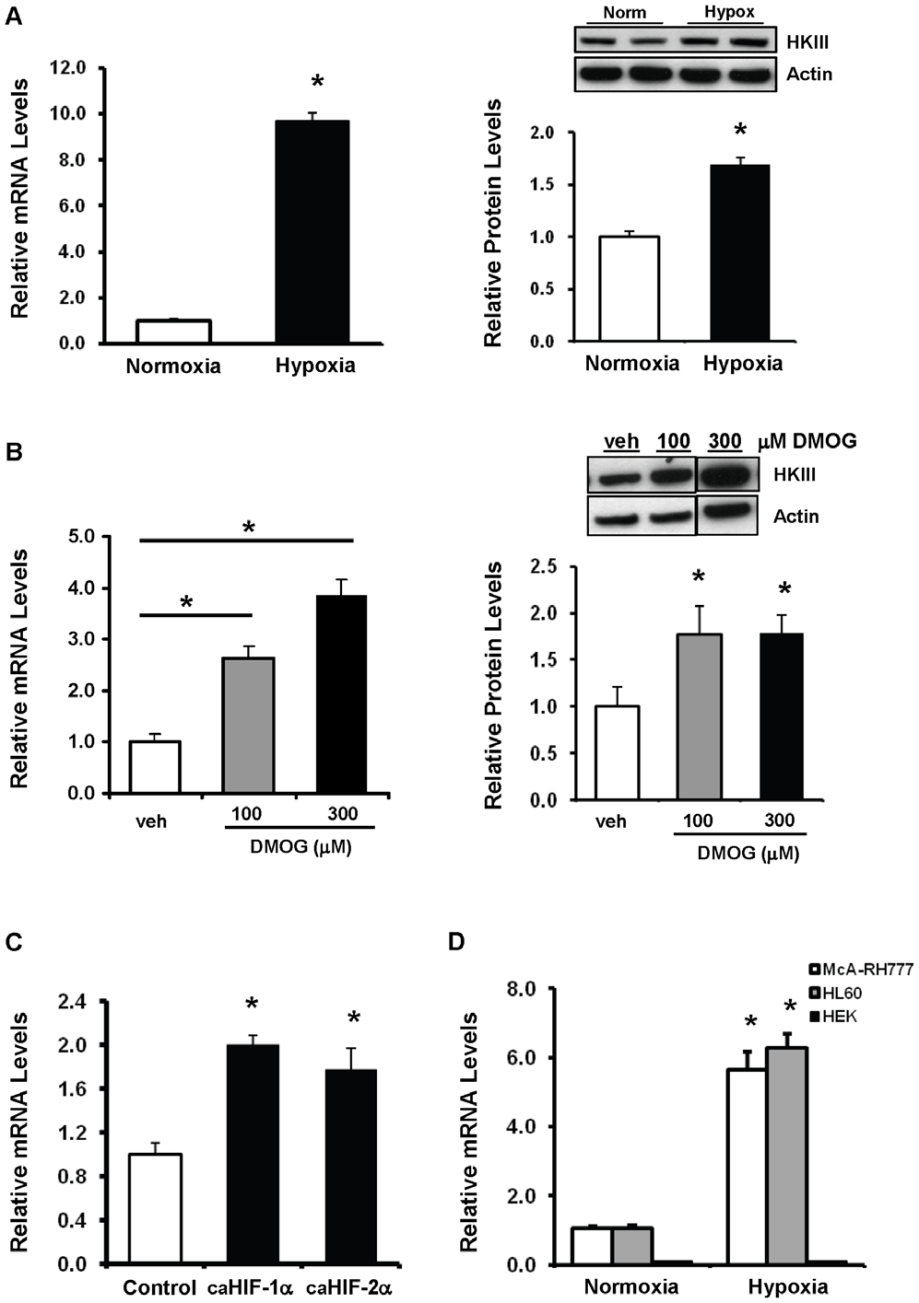
Hypoxia regulates HKIII expression in N1S1 cells via HIF signal transduction. (A) HKIII mRNA and protein levels (left and right panels, respectively) were measured in N1S1 cells cultured under normoxic or hypoxic (1.5% O_2_) conditions for 24 hours. HKIII levels were significantly higher after hypoxia treatment. (B) N1S1 cells were cultured under normoxic conditions for 24 hours with or without a HIF stabilizer (DMOG, 100 and 300 µM). HKIII mRNA and protein levels increased significantly after DMOG treatment. (C) HKIII mRNA levels were significantly increased in N1S1 cells expressing constitutively active (ca) HIF-1α or HIF2α constructs under normoxic conditions, 24 h after transfection. (D) HKIII mRNA levels were significantly increased after hypoxia in another rat hepatoma cell line (McA-RH 7777), as well as a human myeloid leukemia cell line (HL 60). This response was not observed in HEK293 cell which had no detectable HKIII expression under normoxic or hypoxic conditions. *P<0.05 v. control; Data are presented as mean±SEM, N = 3–5 for all groups.

Analysis of the HKIII promoter sequence identified several putative regulatory elements responsive to cAMP, AMPK, or HIF signal transduction, which have been shown to regulate HKII expression [30], [31], [32]. Treatment of Novikoff N1S1 rat hepatoma cells, which have measurable baseline levels of HKIII expression, with the cAMP inducer forskolin or the AMPK activator AICAR did not significantly change HKIII mRNA expression levels (Figure S1A and B). However, HKIII expression significantly increased at the mRNA and protein levels in response to hypoxia (Figure 1A). Hypoxic regulation of HKIII was also demonstrated at the mRNA level in other cell types (Figure 1D).

We then studied whether the regulation of HKIII by hypoxia is through a HIF-dependent pathway. Treatment of N1S1 cells under normoxic conditions with the prolyl hydroxylase inhibitor and HIF stabilizer Dimethyloxallyl glycine (DMOG) resulted in a significant increase in HKIII mRNA and protein levels (Figure 1B). Overexpression of constitutively active (ca) HIF-1α and 2α constructs, also resulted in similar increases in HKIII expression at the mRNA level compared to the vector control (Figure 1C). These constructs contain double P to A point mutations that prevent prolyl hydroxylation and subsequent degradation under normoxic conditions [29]. Overall, the results indicate that regulation of HKIII expression is partially mediated through HIF signaling, which does not appear to be isoform specific. HKII expression served as a positive control for these experiments, and the data are presented in Figure S2A and B).

### N-terminal substrate binding regulates HKIII activity

**Figure 2:**
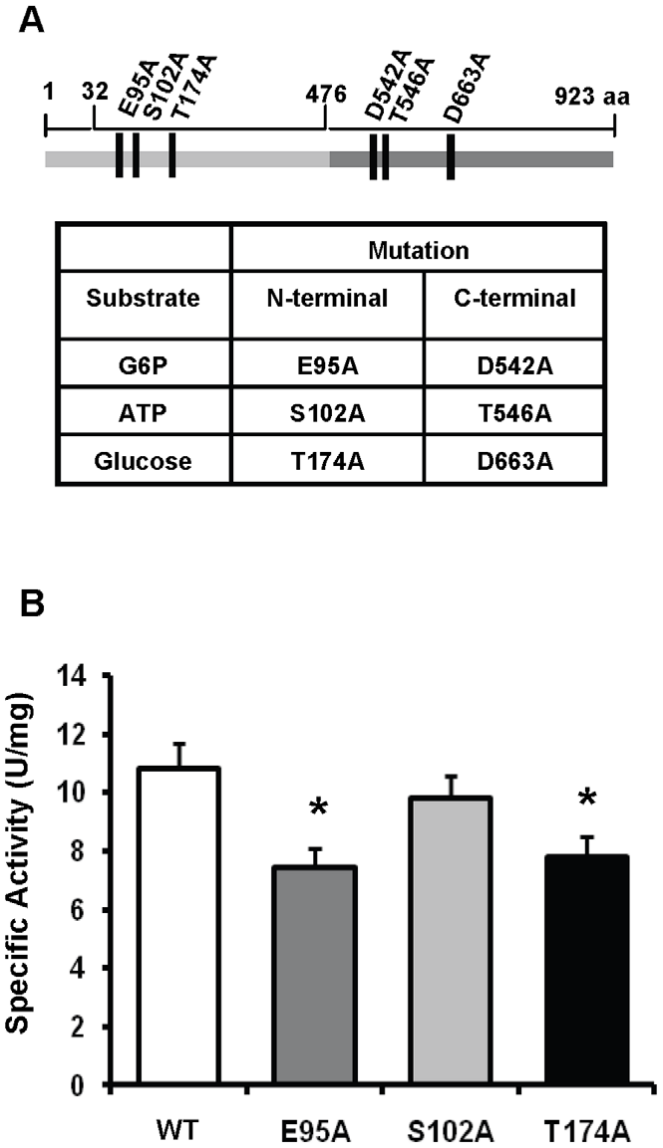
Mutations in putative N-terminal substrate-binding residues of HKIII alter catalytic activity in the C-terminal domain. (A) Six mutant HKIII proteins were generated, each containing a mutation in one of the N-terminal or C-terminal binding sites for G6P, ATP, or glucose. The catalytic activity of each mutant protein was assessed via the G6P/NADPH-coupling assay. (B) Mutations in the putative N-terminal G6P- and glucose-binding sites significantly impaired HKIII specific activity. *P<0.05 vs WT. V*max*  = .13±.010; .09±.009; .12±.009; .098±.009 for Wt, E95A, S102A, and T174A respectively. There was no detectable change in absorbance for the C-terminal mutant constructs. *P<0.05 v. Wt; Data are presented as mean±SEM, N≥3 replicates for each construct.

Glucose binding to the N-terminal domain (N-half) of HKII influences the regulation of the C-half by G6P [17], demonstrating HK regulation at the post-translational level. We studied whether an intramolecular interaction between the two domains also exists in HKIII. The HKIII sequence was aligned with those of HKI and HKII to predict which residues in its N- and C- terminal halves bind glucose, ATP, and G6P. We then generated HKIII constructs with mutations in these putative binding sites (Figure 2A).

Histidine-tagged wildtype and mutant proteins were expressed in a bacterial system and purified on a nickel column, and the enzymatic activity of these constructs was measured using the G6P/NADPH-coupling assay. Mutations in the C-terminal domain abolished glucose phosphorylation, which is consistent with previous reports indicating that the catalytic activity of HKIII is restricted to the C-terminal domain (data not shown) [8], [19], [33]. Mutations in the putative N-terminal glucose- and G6P-binding sites reduced the specific activity of the protein (Figure 2B), but the putative N-terminal ATP-binding site mutation did not. Collectively, these results suggest that inhibition of glucose or G6P binding, but not ATP binding, in the N-terminal domain reduces the catalytic activity of the C-terminal domain. Thus, there is functional interaction between the N- and C-halves of HKIII.

### HKIII overexpression protects against oxidant-induced cell death

**Figure 3:**
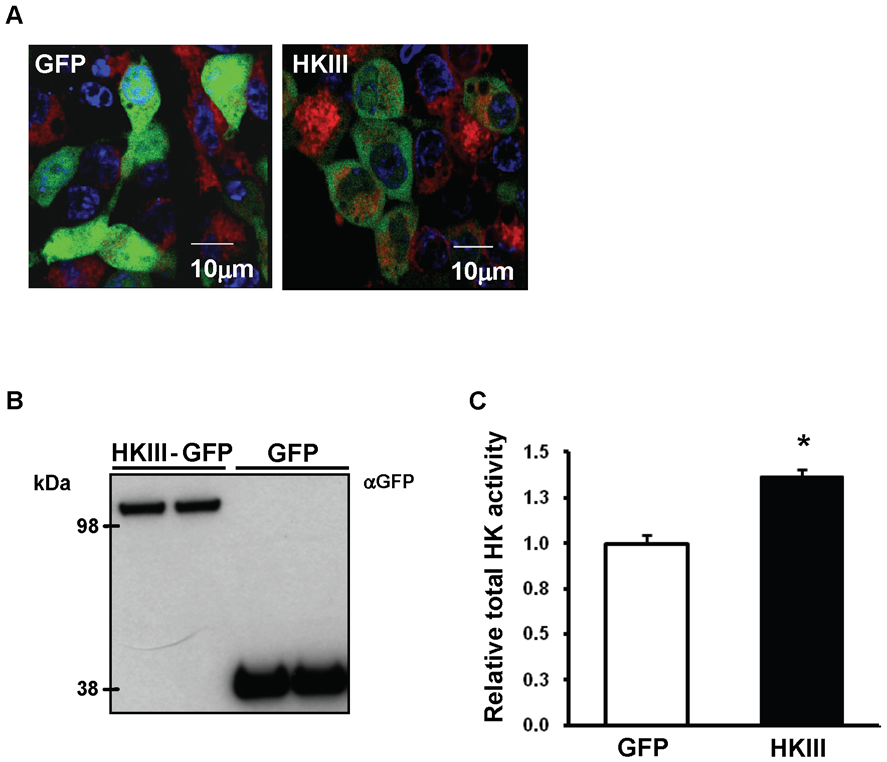
Overexpression of HKIII in HEK293 cells. (A) Confocal microscopy images demonstrate successful transfection of HEK293 cells with the GFP and HKIII-GFP vector plasmids. HKIII-GFP does not appear cytosolic with no observed localization to any subcellular compartment. The cells were stained with TMRE (red) to identify mitochondria, and nuclei were labeled with Hoechst (blue). (B) Overexpression was confirmed by Western blots of cell lysates with a GFP antibody. (C) Total hexokinase activity was significantly increased in cells overexpressing HKIII compared to GFP control.

Previous studies have shown that HKI and II protect cells from oxidant-induced cell death [27], [34]. We next studied the potential role of HKIII in cytoprotection, using a similar model of H_2_O_2_ induced cell death in HEK293 (HEK) cells [27]. Cells were transfected with plasmids coding for GFP or HKIII-GFP expression, and overexpression was confirmed via confocal fluorescent microscopy and Western blot (Figure 3A and B). Furthermore, overexpression of the HKIII-GFP construct resulted in a significant increase in total hexokinase activity compared to GFP (Figure 3C).

**Figure 4:**
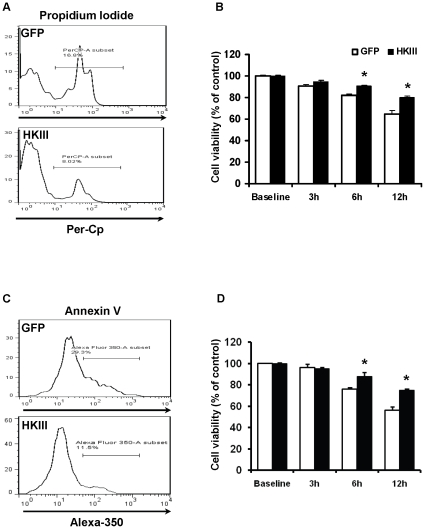
Overexpression of HKIII protects against oxidant induced cell death. HEK cells overexpressing HKIII or GFP were treated with 500 µM of H_2_O_2_, and assayed for cell death using propidium iodide (PI) staining as an indicator of compromised membrane integrity and Annexin V as a marker of apoptosis. (A) Representative flow output demonstrating a reduction in the number of PI positive cells after treatment with H_2_O_2_ (500 µM, 6 h) in samples overexpressing HKIII v GFP. (B) Cell viability was significantly increased in cells overexpressing HKIII compared to GFP after exposure to 500 µM H_2_O_2_ at the 6 and 12 h time points, as determined by propidium iodide (PI) exclusion. (C) Representative flow output demonstrating a reduction in the number of cells positive for the apoptotic marker Annexin V after treatment with H_2_O_2_ (500 µM, 6 h) in samples overexpressing HKIII v GFP. (D) HKIII overexpression resulted in decrease in apoptotic cell death after exposure to 500 µM H_2_O_2_ at the 6 and 12 h time points, as determined by AnnexinV. Cell counts were normalized to counts obtained without H_2_O_2_ treatment. *P<0.05 vs GFP. Data are presented as mean±SEM. N = 3–6.

Thirty-six to forty-eight hours after transfection, cells were serum starved overnight in basal MEM media, and then exposed to oxidative stress using exogenous H_2_O_2_. Cell viability was assessed using propidium iodide exclusion and Annexin V to evaluate necrotic and apoptotic cell death, respectively. Both assays demonstrate that survival was higher in cells transfected with the HKIII-GFP plasmid than in GFP transfected controls (Figure 4 A–D). These results indicate that HKIII protects against oxidant-induced cell death.

### HKIII overexpression increases ATP levels and reduces ROS production

**Figure 5:**
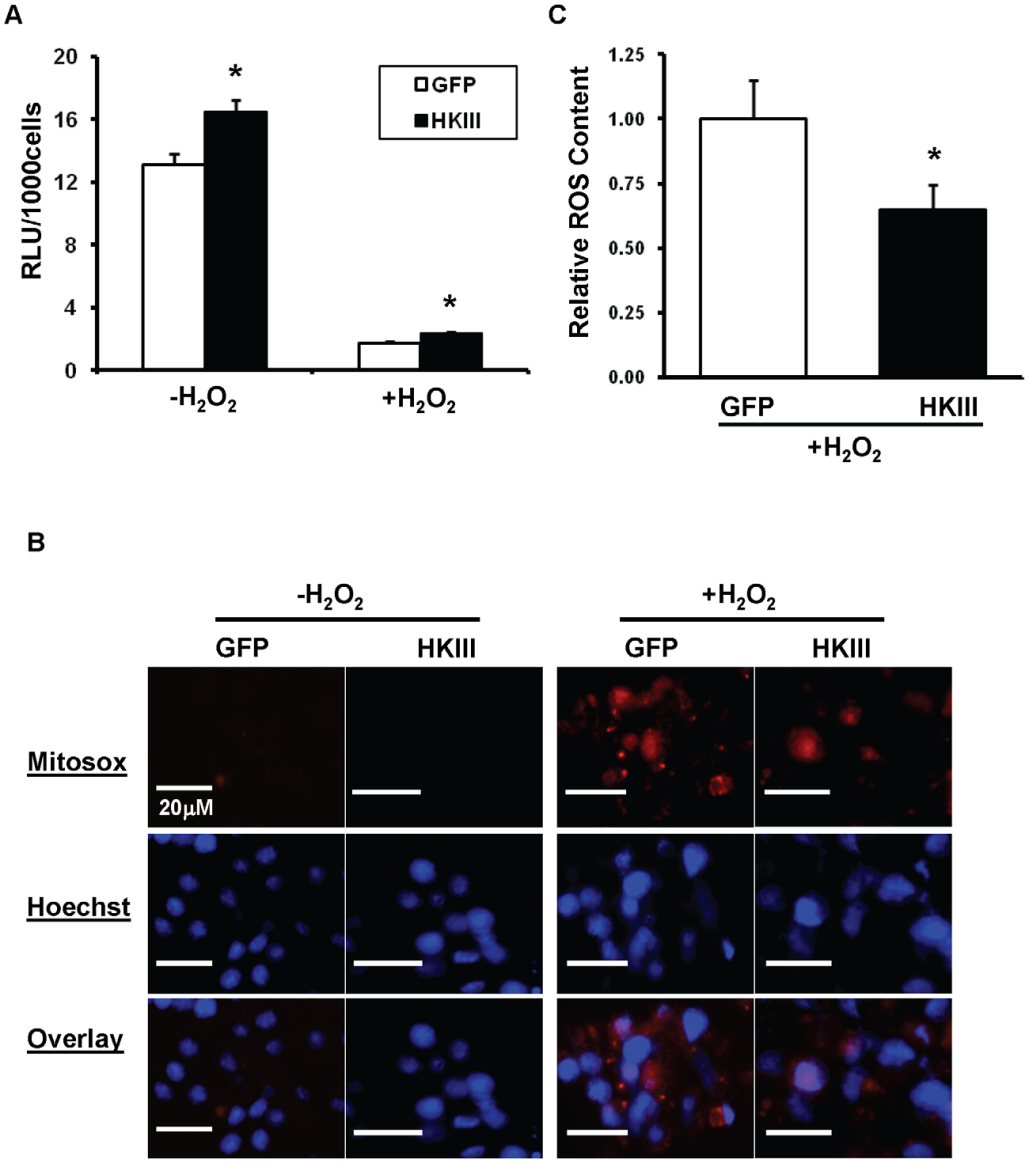
Overexpression of HKIII increases ATP levels and reduces oxidant-induced ROS levels. (A) ATP levels were significantly higher in cells overexpressing HKIII both at baseline and after treatment with exogenous H_2_O_2_ (500 µM, 6 h). Cells were incubated overnight in serum free basal MEM media, and ATP levels were determined via the Cell Titer Glo assay, with luminescence (RLU) normalized to the number of viable cells. N = 6. (B) HEK293 cells were transfected with HKIII-GFP or GFP, treated with or without 500 µM H_2_O_2_ (3 h), and then ROS levels were measured with a fluorescent marker of superoxide production (MitoSox, red). Nuclei are labeled with Hoechst (blue), and the GFP overlay for these images is presented in the supplemental material. (C) HKIII overexpression was associated with significantly lower ROS levels in H_2_O_2_-treated cells. (N = 3, ROS levels for each sample represent the average fluorescent intensity from 5 separate fields, each normalized to cell number). *P<0.05 vs GFP. Data are presented as mean±SEM.

Glucose metabolism is necessary to maintain cellular ATP levels which is vital to cell viability [35]. To evaluate the effect of HKIII on cellular energy production, we measured ATP levels in HEK cells transfected with HKIII-GFP or GFP plasmid. Cells were serum starved overnight in basal MEM media, and then treated with 500 µM H2O2 before the assessment of ATP levels. Cells overexpressing HKIII-GFP had significantly higher ATP levels at baseline and after exposure to H_2_O_2_, compared with GFP controls (Figure 5A).

Increased ROS production can also induce cell death, and we have previously shown that the overexpression of HKI or HKII reduces cellular ROS levels [27]. Here, we evaluated the potential influence of HKIII on ROS production. Under normal culture conditions, ROS levels in cells transfected with HKIII-GFP or GFP were not readily detected using the fluorescent mitochondrial superoxide indicator MitoSox (Figure 5B); however, ROS production after exposure to exogenous H_2_O_2_ was significantly lower in HKIII-GFP overexpressing cells compared to GFP controls (Figures 5B and C, S3A and B). Collectively, these results suggest that the reduction in cell death associated with HKIII overexpression could evolve from an increase in ATP levels or a decline in ROS production.

### HKIII overexpression leads to increased mitochondrial membrane potential and mitochondrial biogenesis

**Figure 6:**
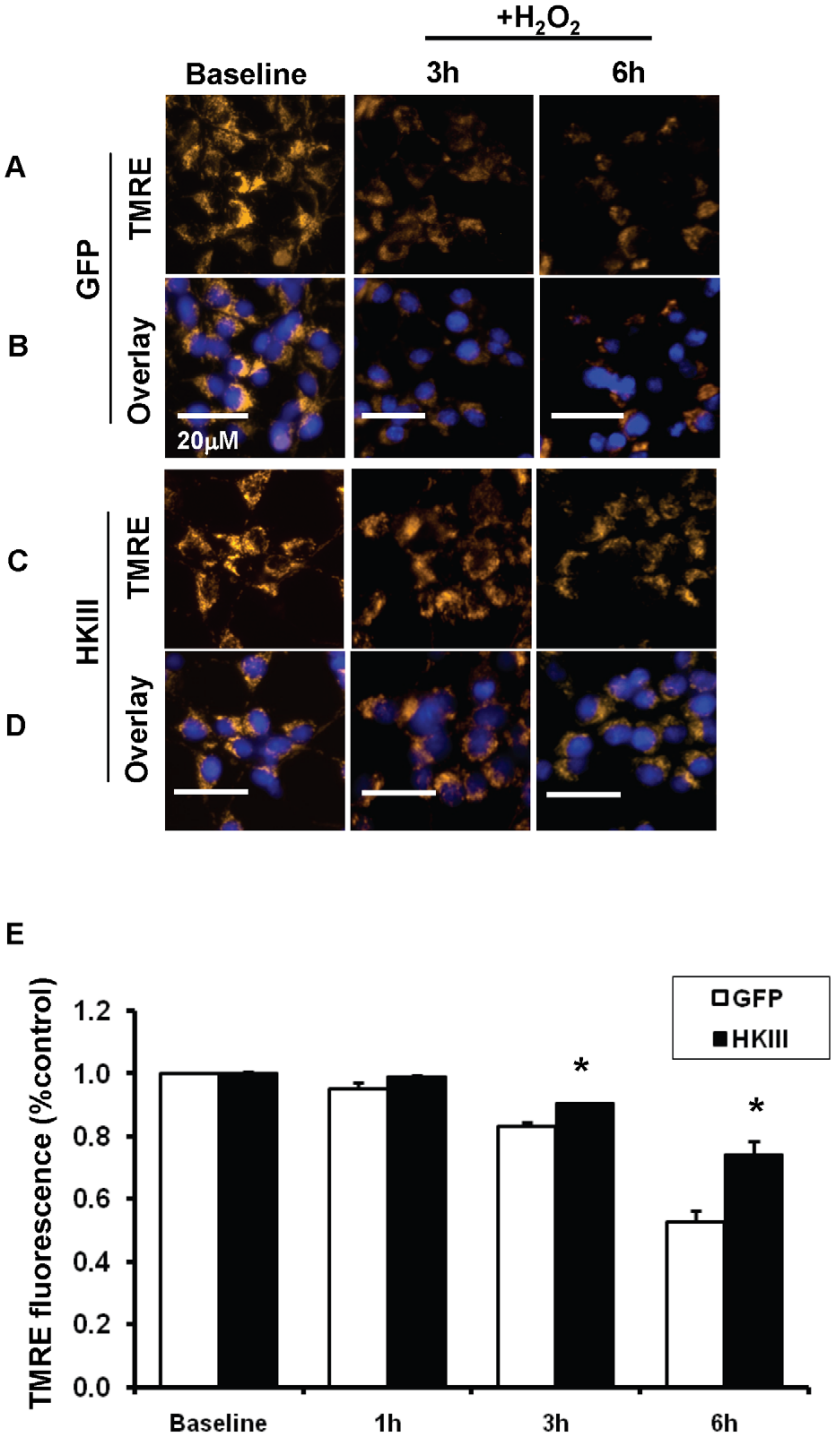
HKIII overexpression preserves mitochondrial membrane potential in cells exposed to oxidant stress. Mitochondrial membrane potential was assessed in HEK cells overexpressing HKIII or GFP after treatment with H_2_O_2_, using the fluorescent dye tetramethylrhodamine ethyl ester TMRE (orange). (A) Fluorescent images taken 3 and 6 h after exposure to 500 µM of H_2_O_2_ demonstrate a pronounced reduction in TMRE fluorescence over time in cells overexpressing GFP that was attenuated in (C) cells overexpressing HKIII. TMRE images are overlayed with the Hoechst nuclear stain (blue) for (B) GFP and (D) HKIII. The fluorescent GFP overlay for each image is presented in the supplemental material. (E) TMRE levels, quantified by flow cytometry, were significantly higher in cells overexpressing HKIII v GFP control 3 and 6 h after treatment with 500 µM of H_2_O_2_. N = 3–6. *P<0.05 vs GFP. Data are presented as mean±SEM.

The increased energy reserve and reduced ROS levels observed in stressed cells overexpressing HKIII suggest that HKIII may have an impact on the mitochondria. Previous studies have shown that HKI and II attenuated the loss of mitochondrial membrane potential in cells exposed to oxidative stress [27]. We next studied the effects of HKIII overexpression on membrane potential using TMRE as previously described [27]. Overexpression of HKIII did not impact overall TMRE fluorescence at baseline; however TMRE fluorescence was significantly higher in cells overexpressing HKIII-GFP after exposure to oxidative stress compared to GFP controls (Figures 6A-E, S4A and B).

**Figure 7:**
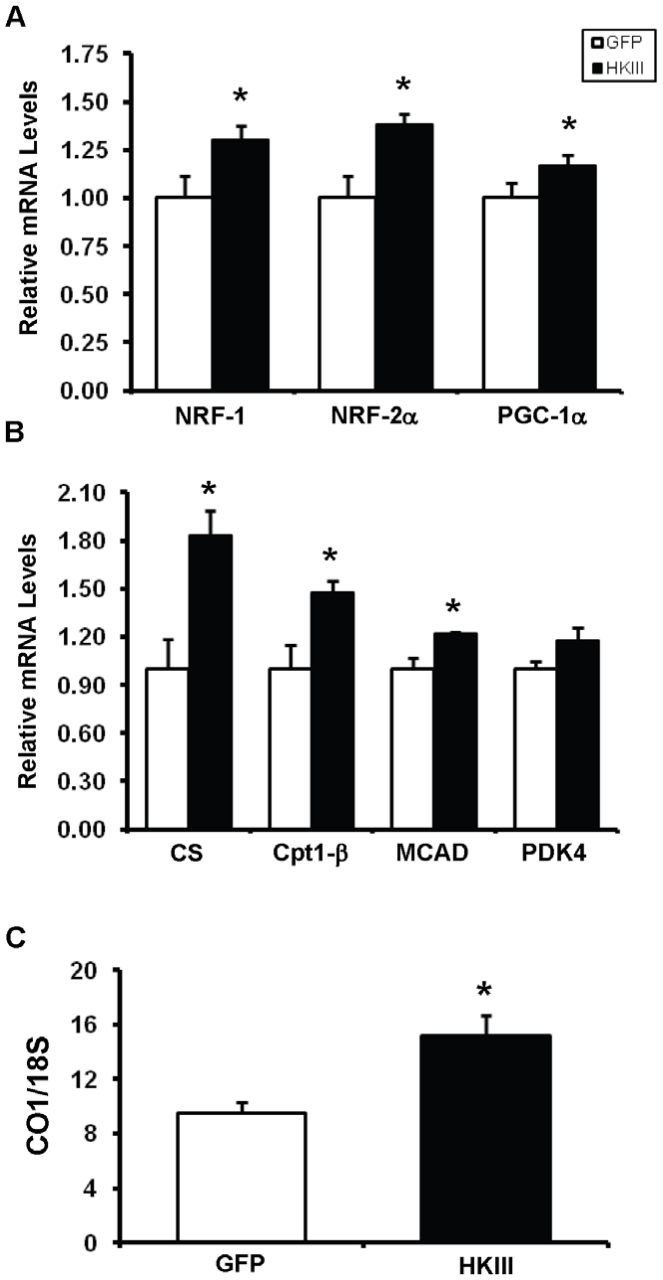
Overexpression of HKIII increases expression of markers associated with mitochondrial biogenesis and mitochondrial DNA content. (A) HKIII overexpression lead to significant increases in the expression of NRF-1, NRF-2α, and PGC-1α, which regulate mitochondrial biogenesis. (B) Genes encoding regulatory elements of the TCA cycle, fatty acid transport, and β-oxidation were also upregulated after HKIII overexpression. Data represent transcript levels first normalized to an internal housekeeping control and presented relative to GFP expression. (C) HEK293 cells were transfected with HKIII-GFP or GFP; 48 hours later, changes in mitochondrial DNA content were determined by measuring the levels of the mitochondrial encoded gene CO1 and the nuclear encoded 18S gene. Overexpression of HKIII significantly increased the ratio of mitochondrial encoded to nuclear-encoded DNA. *P<0.05. Data are presented as mean±SEM, N = 3. CO1: cytochrome c oxidase subunit 1; Cpt1-β: Carnitine O-palmitoyltransferase I, muscle isoform; CS: citrate synthase; MCAD: Medium-chain acyl-CoA dehydrogenase; NRF: Nuclear Regulatory Factor 1,2α; PDK4: pyruvate dehydrogenase kinase 4; PGC-1α: Peroxisome proliferator-activated receptor gamma coactivator-1 alpha.

Studies have also shown that elevated glucose metabolism is associated with increased mitochondrial biogenesis [36], [37], so we compared the expression of genes known to play a role in mitochondrial biogenesis and the overall mitochondrial DNA content in HEK293 cells transfected with HKIII-GFP or GFP. Overexpression of HKIII increased mRNA levels of PGC-1α and the nuclear regulatory factors (NRF) 1 and 2α (Figure 7A), which regulate the expression of nuclear genes involved in mitochondrial biogenesis. HKIII overexpression also induced expression of several genes involved in the TCA cycle, fatty acid transport, and β-oxidation (Figure 7B). Total mitochondrial DNA content was also higher in cells that overexpressed HKIII-GFP than in GFP-overexpressing cells (Figure 7C). Thus, the overexpression of HKIII appears to increase mitochondrial biogenesis. This process could also contribute to the cytoprotective effects of HKIII.

### The N-terminal 32-amino-acid domain of HKIII is required for proper protein expression

**Figure 8:**
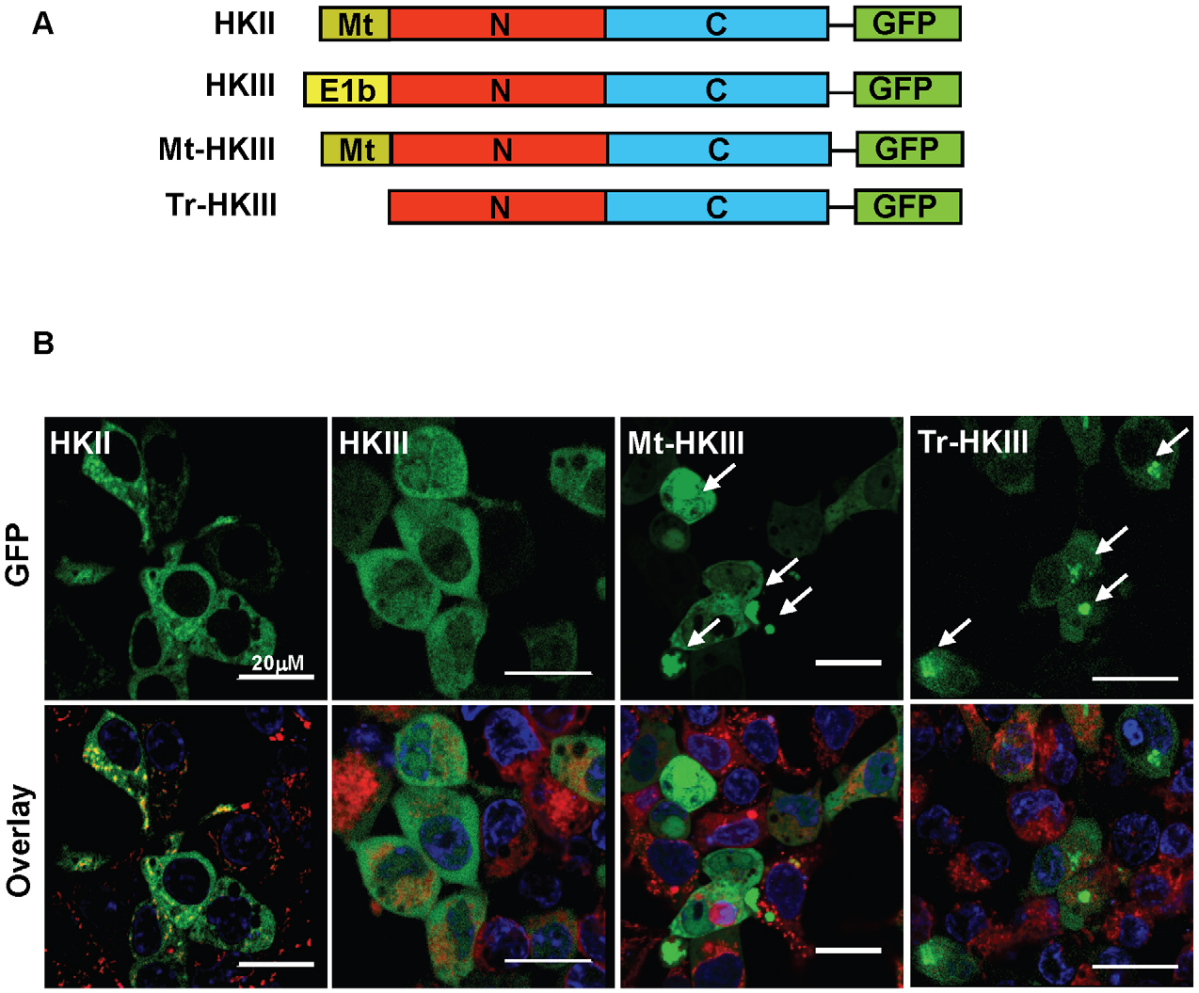
Removal of the N-terminal 32 amino acids of HKIII causes protein aggregation. (A) Plasmids coding for the expression of the full-length HKII (HKII); full length HKIII (HKIII), a chimeric protein replacing the 32amino acid N-terminal region of HKIII (encoded by exon 1b) with the 21aa mitochondrial-targeting sequence of HKII (Mt-HKIII), and a truncated version that lacked the N-terminal 32aa region encoded by exon 1b (Tr-HKIII). Each construct also coded for GFP expression at the C-terminus. (B) Live cell confocal images of HEK cells after transfection with the indicated constructs. Overexpression of HKII resulted in co-localization with the mitochondria membrane marker TMRE (red). Co-localization was not observed with overexpression of the HKIII or Mt-HKIII constructs. Overexpression of Mt-HKIII or the truncated HKIII constructed resulted in aggregate formation (white arrows). Nuclei were labeled with Hoechst (blue).

Previous studies have shown that the binding of HKI and HKII to mitochondria protects against cell death [27]. Thus, we investigated whether the protective effects of HKIII, which is a cytoplasmic protein, could be enhanced by replacing its N-terminal 32 amino acids (encoded by exon 1b of HKIII) with the 21-amino-acid mitochondrial-targeting (Mt) sequence of HKII (encoded by exon 1 of HKII) (Figure 8A). Overexpression of the chimeric Mt-HKIII protein (containing the HKII mitochondrial-targeting sequence in place of the N-terminal 32 amino acids of HKIII) did not lead to co-localization with the mitochondria, which was observed with overexpression of HKII (Figure 8B) [27]. In addition, overexpression of the mitochondrial targeted chimera (Mt-HKIII) led to the formation of protein aggregates (Figure 8B). To determine whether aggregation was caused by the removal of the native HKIII 32amino acid sequence, or by the addition of the mitochondrial-targeting sequence of HKII, we deleted the native sequence only (Figure 8A). Overexpression of this truncated construct (Tr-HKIII) also caused protein aggregation (Figure 8B), suggesting that the N-terminal 32 amino acids of HKIII are essential for proper folding of the protein. Consistent with these findings, overexpression of the C-terminal domain of HKIII formed aggregates, whereas the N-terminal domain was soluble, and its cytosolic expression was similar to that of the full-length protein (Figure S5A and B).

In an attempt to identify specific N-terminal HKIII residues required to maintain solubility, we progressively deleted 8-amino-acid sequences in this region, and expressed these mutants in HEK293 cells (Figure S6A). The overexpressed proteins were soluble; indicating that partial truncation of this N-terminal region does not result in aggregate formation (Figure S6B). Western blot demonstrating protein expression in HEK293 cell lysates is shown for all constructs in Figure S7A. Overall, these results suggest that the N-terminal 32-amino-acid sequence of HKIII is necessary to maintain solubility and proper expression of the protein.

## Discussion

Three 100-kDa HK isozymes are expressed in mammalian tissue [7], [8], [27], [38], [39]. The regulation, localization, activity, and cytoprotective capacity of HKI and HKII have been extensively studied. [10]–[17], [21], [22], [27]. However, the regulation of HKIII and its potential cytoprotective role are not well characterized. Here, we show that: 1) HKIII is regulated by hypoxia through a HIF-dependent pathway, 2) glucose and G6P binding to the N-terminal domain regulates the catalytic activity of the C-terminal domain, 3) overexpression of HKIII protects cells from oxidant-induced cell death, and 4) HKIII overexpression increases cellular ATP levels, reduces ROS production, preserves mitochondria membrane potential, and promotes the expression of genes that regulate mitochondrial biogenesis. The latter results suggest that the protective effects of HKIII may evolve through a decline in ROS levels and increases in ATP and mitochondrial biogenesis. Our results also demonstrate that the 32 amino acids encoded by exon 1b of HKIII and located at the protein's N-terminus appear to be essential for proper folding, because deletion of these residues led to protein aggregation.

Previous studies have identified distinct differences between the regulation of HKI and HKII [7], [8]. Analysis of the HKI promoter, as well as the ubiquitous tissue expression of this isoform, indicate that HKI is constitutively expressed, while HKII expression is tissue specific and regulated by stimuli such as insulin and hypoxia [7], [30], [31], [32], [39], [40]. HKIII is primarily expressed in the lung, kidney, and liver [7], [9], [41], and an Oct-1 binding site is necessary for basal HKIII expression [9], but little else is known about the regulation of HKIII activity. Sequence analysis of the HKIII promoter identified several putative regulatory elements that may respond to cAMP, AMPK, or HIF signal transduction. Pharmacological stimulation with cAMP or AMPK activators (forskolin and AICAR, respectively) had no measurable effect on HKIII mRNA expression, but cellular HKIII expression was significantly increased by hypoxia through a HIF-dependent pathway. These findings are the first to demonstrate that HKIII expression is regulated by a specific signaling pathway, and identifies a likely role for this isozyme in the cellular response to the metabolic stresses induced by hypoxia. Furthermore, the inducible expression of HKII and III suggests that these enzymes have a role in adaptive metabolic responses to changes in the cellular environment.

Previous reports also indicate that the two domains of HKI and HKII do not function independently. Here, we demonstrate that mutation of the glucose- or G6P-binding sites in the N-half of HKIII impairs catalysis in the C-half. Thus, the activity of HKIII appears to be governed, in part, by functional interactions between the two domains. Previous studies on HKI demonstrate a cooperative effect of glucose binding in the N-half to subsequent binding in the C-half [42], [43]. It is possible that a similar intramolecular interaction occurs in HKIII, and reduction in N-half glucose binding results in diminished C-half binding, and an overall reduction in activity; however, other possibilities cannot be excluded. We also found that a mutation in the putative N-terminal G6P binding site reduced HKIII activity. This observation (i.e., that mutation of an inhibitory site would decrease enzyme activity) was unexpected and may have been caused by a structural change that adversely affected glucose binding in the N-half. Furthermore, mutation of the N-terminal ATP binding site did not alter the catalytic activity of the enzyme, which suggests that either the mutated residue was not essential for N-terminal ATP binding, or ATP binding in the N-half of HKIII does not regulate the activity of the C-half. Further enzymatic analysis along with continued work to generate a crystal structure for HKIII will help clarify the effects of N-terminal substrate binding on HKIII activity.

The protective effects of HKI and HKII are believed to occur through changes in the glucose-phosphorylation and mitochondrial-binding activity of the enzymes [27]. Since HKIII does not bind to mitochondria, we proposed that its protective effects are due to: 1) an increase in glucose phosphorylation, energy production and mitochondrial biogenesis, and 2) a reduction in cellular ROS levels. Our results demonstrate that overexpression of HKIII results in an increase in cellular ATP levels and mitochondrial biogenesis, and a decrease in oxidant induced ROS production. Although these results do not demonstrate a cause and effect relationship between these factors and cell death, they implicate their possible role in this process.

The binding of HKI and HKII to the mitochondria is physiologically significant, because it contributes to their cytoprotective effects, and dislocation of HKI and HKII to the cytoplasm is associated with higher rates of cell death [26], [27], [44]. Mitochondrial binding occurs through a 21-amino-acid, N-terminal sequence encoded by exon 1 of the HKI and HKII genes, which is predicted to form a hydrophobic alpha-helix that binds to the outer membrane of the mitochondria. In this study, we investigated whether the protective effects of HKIII could be enhanced by replacing the N-terminal 32 amino acids of HKIII (encoded by exon 1b) with the 21-amino-acid mitochondrial-targeting sequence of HKII (encoded by exon 1). Our results indicate that the sequence in HKIII, encoded by exon 1b, is critical for protein folding, since its deletion or its replacement with the mitochondrial-targeting sequence of HKII resulted in the formation of intracellular aggregates. The sequences encoded by exon 1 are unique to the 100-kDa mammalian HKs and they are not present in the 50-kDa HKs expressed in yeast and other organisms. In 100-kDa enzymes, the N-half (encoded by exons 2 through half of exon 10) and the C-half (encoded by exons 10-18) form the catalytic and regulatory portion of the proteins, while exon 1 is not part of the duplication process and encodes unique sequences. We showed that unlike HKI and HKII, this region of HKIII is needed for proper folding of the protein and that its deletion makes the enzyme insoluble, leading to aggregate formation.

In summary, the results reported here are the first to describe the regulation of HKIII expression and activity and to characterize the role of HKIII in cytoprotection. HKIII expression is regulated by hypoxia through a HIF-dependent signal transduction pathway, and the activity of HKIII is also regulated at the protein level by the influence of N-terminal substrate binding on C-terminal catalysis. HKIII overexpression promotes cell survival in response to oxidative stress, perhaps by increasing cellular ATP levels, decreasing ROS production, and promoting mitochondrial biogenesis, and the sequence encoded by exon 1b of HKIII is critical for proper protein folding, whereas the corresponding sequences in HKI and HKII function as mitochondrial-targeting signals. Collectively, these observations provide insights into the specific role of the HKIII isozyme in cell metabolism and function.

## Supporting Information

Table S1 Primer Sequences used for PCR Amplification
(0.60 MB TIF)

Figure S1 HKIII levels do not change in N1S1 cells after cAMP or AMPK activation. HKIII mRNA expression was measured in N1S1 cells cultured for 24 hours with or without (A) 5 microM forskolin (an inducer of cAMP production) or (B) 1 mM AICAR (an activator of AMPK). HKIII mRNA levels in treated and untreated cells were similar. Data are presented as mean+SEM, N = 3 for all groups.
(0.52 MB TIF)

Figure S2 Hypoxia regulates HKII expression in N1S1 cells. (A) HKII mRNA levels were measured in N1S1 cells cultured under normoxic or hypoxic (1.5% O2) conditions for 24 hours. HKII levels were significantly increased after hypoxia treatment. (B) N1S1 cells were cultured under normoxic conditions for 24 hours with or without a HIF stabilizer (DMOG, 100 and 300 µM). HKII mRNA levels increased significantly after DMOG treatment. (C) Comparative mRNA expression levels of the HKII and HKIII isoforms in N1S1 cells cultured under normoxic or hypoxic (1.5% O2) conditions for 24 hours, demonstrating the higher levels of HKII expression in this cell type. *P<0.05 v. control; Data are presented as mean±SEM, N = 3–5 for all groups.
(0.45 MB TIF)

Figure S3 Overexpression of GFP and HKIII-GFP in HEK cells labeled with Mitosox. (A) GFP fluorescent overlay for the HEK cell images displayed in figure 5B, demonstrating the overexpression of the GFP and HKIII-GFP constructs in cells treated with or without 500 µM H2O2 (3 h), and then incubated with (MitoSox). Cell nuclei are labeled with Hoechst (blue).
(2.61 MB TIF)

Figure S4 Overexpression of GFP and HKIII-GFP in HEK cells labeled with TMRE. (A) GFP fluorescent overlay for the HEK cell images displayed in figure 6 A-D, demonstrating the overexpression of the GFP (top panels) and HKIII-GFP (bottom panels) constructs in cells loaded with TMRE(orange) prior to the treatment condition indicated. Cell nuclei are labeled with Hoechst (blue).
(5.19 MB TIF)

Figure S5 Overexpression of the N and C-terminal domains of HKIII in HEK293 cells. (A) Plasmid constructs coding for the expression of the N and C-terminal domains of HKIII. Each construct also coded for GFP expression at the C-terminus. (B) Live cell confocal images of HEK cells after transfection with the indicated constructs reveals soluble expression of the N-terminal half of HKIII (NHKIII) similar to the full length HKIII construct, while overexpression of the C-terminal domain resulted in aggregate formation. The cells were incubated with TMRE (red) to identify mitochondria, and nuclei were labeled with Hoechst (blue).
(2.89 MB TIF)

Figure S6 Progressive deletion of 8-amino-acid segments from the N-terminus of HKIII do not cause protein aggregation. (A) Plasmids expressing truncated versions of HKIII that lacked the first 8 (HKIII-8), 16 (HKIII-16), or 24 (HKIII-24) amino acids of the N-terminal sequence encoded by exon 1b were constructed and expressed in HEK293 cells; each construct also coded for GFP expression at the C-terminus. (B) Live cell confocal images of HEK cells after transfection with the indicated constructs showed that the full-length protein and each of the three serial truncation constructs were expressed in the cytoplasm and did not form cellular aggregates. The cells were incubated with TMRE (red) to identify mitochondria, and nuclei were label with Hoechst (blue).
(3.35 MB TIF)

Figure S7 Western blot for protein expression for the HKIII constructs. (A) Western blot of cell lysates from HEK293 cells overexpressing the indicated constructs. For each construct, 1.5 µg of plasmid DNA was transfected into each well using Lipofectamine 2000 as described.
(1.33 MB TIF)

## References

[1] Bell GI, Burant CF, Takeda J, Gould GW (1993). Structure and function of mammalian facilitative sugar transporters.. J Biol Chem.

[2] Gould GW, Holman GD (1993). The glucose transporter family: structure, function and tissue-specific expression.. Biochem J.

[3] Mueckler M (1994). Facilitative glucose transporters.. Eur J Biochem.

[4] Printz RL, Osawa H, Ardehali H, Koch S, Granner DK (1997). Hexokinase II gene: structure, regulation and promoter organization.. Biochem Soc Trans.

[5] Middleton RJ (1990). Hexokinases and glucokinases.. Biochem Soc Trans.

[6] Ureta T (1982). The comparative isozymology of vertebrate hexokinases.. Comp Biochem Physiol B.

[7] Wilson JE (2003). Isozymes of mammalian hexokinase: structure, subcellular localization and metabolic function.. J Exp Biol.

[8] Cardenas ML, Cornish-Bowden A, Ureta T (1998). Evolution and regulatory role of the hexokinases.. Biochim Biophys Acta.

[9] Sebastian S, Edassery S, Wilson JE (2001). The human gene for the type III isozyme of hexokinase: structure, basal promoter, and evolution.. Arch Biochem Biophys.

[10] Arora KK, Filburn CR, Pedersen PL (1991). Glucose phosphorylation. Site-directed mutations which impair the catalytic function of hexokinase.. J Biol Chem.

[11] Arora KK, Filburn CR, Pedersen PL (1993). Structure/function relationships in hexokinase. Site-directed mutational analyses and characterization of overexpressed fragments implicate different functions for the N- and C-terminal halves of the enzyme.. J Biol Chem.

[12] Baijal M, Wilson JE (1992). Functional consequences of mutation of highly conserved serine residues, found at equivalent positions in the N- and C-terminal domains of mammalian hexokinases.. Arch Biochem Biophys.

[13] Magnani M, Bianchi M, Casabianca A, Stocchi V, Daniele A (1992). A recombinant human ‘mini’-hexokinase is catalytically active and regulated by hexose 6-phosphates.. Biochem J.

[14] Nemat-Gorgani M, Wilson JE (1986). Rat brain hexokinase: location of the substrate nucleotide binding site in a structural domain at the C-terminus of the enzyme.. Arch Biochem Biophys.

[15] Schirch DM, Wilson JE (1987). Rat brain hexokinase: location of the substrate hexose binding site in a structural domain at the C-terminus of the enzyme.. Arch Biochem Biophys.

[16] White TK, Wilson JE (1987). Rat brain hexokinase: location of the allosteric regulatory site in a structural domain at the N-terminus of the enzyme.. Arch Biochem Biophys.

[17] Ardehali H, Printz RL, Whitesell RR, May JM, Granner DK (1999). Functional interaction between the N- and C-terminal halves of human hexokinase II.. J Biol Chem.

[18] Ardehali H, Yano Y, Printz RL, Koch S, Whitesell RR (1996). Functional organization of mammalian hexokinase II. Retention of catalytic and regulatory functions in both the NH2- and COOH-terminal halves.. J Biol Chem.

[19] Tsai HJ (1999). Functional organization and evolution of mammalian hexokinases: mutations that caused the loss of catalytic activity in N-terminal halves of type I and type III isozymes.. Arch Biochem Biophys.

[20] Tsai HJ, Wilson JE (1997). Functional organization of mammalian hexokinases: characterization of the rat type III isozyme and its chimeric forms, constructed with the N- and C-terminal halves of the type I and type II isozymes.. Arch Biochem Biophys.

[21] Sui D, Wilson JE (1997). Structural determinants for the intracellular localization of the isozymes of mammalian hexokinase: intracellular localization of fusion constructs incorporating structural elements from the hexokinase isozymes and the green fluorescent protein.. Arch Biochem Biophys.

[22] Xie GC, Wilson JE (1988). Rat brain hexokinase: the hydrophobic N-terminus of the mitochondrially bound enzyme is inserted in the lipid bilayer.. Arch Biochem Biophys.

[23] Preller A, Wilson JE (1992). Localization of the type III isozyme of hexokinase at the nuclear periphery.. Arch Biochem Biophys.

[24] Azoulay-Zohar H, Israelson A, Abu-Hamad S, Shoshan-Barmatz V (2004). In self-defence: hexokinase promotes voltage-dependent anion channel closure and prevents mitochondria-mediated apoptotic cell death.. Biochem J.

[25] Halestrap AP, McStay GP, Clarke SJ (2002). The permeability transition pore complex: another view.. Biochimie.

[26] Pastorino JG, Shulga N, Hoek JB (2002). Mitochondrial binding of hexokinase II inhibits Bax-induced cytochrome c release and apoptosis.. J Biol Chem.

[27] Sun L, Shukair S, Naik TJ, Moazed F, Ardehali H (2008). Glucose phosphorylation and mitochondrial binding are required for the protective effects of hexokinases I and II.. Mol Cell Biol.

[28] Narayanan PK, Hart T, Elcock F, Zhang C, Hahn L (2003). Troglitazone-induced intracellular oxidative stress in rat hepatoma cells: a flow cytometric assessment.. Cytometry A.

[29] Kulshreshtha R, Ferracin M, Wojcik SE, Garzon R, Alder H (2007). A microRNA signature of hypoxia.. Mol Cell Biol.

[30] Ahmad A, Ahmad S, Schneider BK, Allen CB, Chang LY (2002). Elevated expression of hexokinase II protects human lung epithelial-like A549 cells against oxidative injury.. Am J Physiol Lung Cell Mol Physiol.

[31] Holmes BF, Kurth-Kraczek EJ, Winder WW (1999). Chronic activation of 5′-AMP-activated protein kinase increases GLUT-4, hexokinase, and glycogen in muscle.. J Appl Physiol.

[32] Rempel A, Mathupala SP, Griffin CA, Hawkins AL, Pedersen PL (1996). Glucose catabolism in cancer cells: amplification of the gene encoding type II hexokinase.. Cancer Res.

[33] Palma F, Agostini D, Polidori E, Stocchi V (2002). The overexpressed hexahistidine-tagged human hexokinase type III is inhibited by D-glucose.. Prep Biochem Biotechnol.

[34] Pastorino JG, Hoek JB (2003). Hexokinase II: the integration of energy metabolism and control of apoptosis.. Curr Med Chem.

[35] Bryson JM, Coy PE, Gottlob K, Hay N, Robey RB (2002). Increased hexokinase activity, of either ectopic or endogenous origin, protects renal epithelial cells against acute oxidant-induced cell death.. J Biol Chem.

[36] Holloszy JO (2008). Regulation by exercise of skeletal muscle content of mitochondria and GLUT4.. J Physiol Pharmacol.

[37] Riordan MM, Weiss EP, Meyer TE, Ehsani AA, Racette SB (2008). The effects of caloric restriction- and exercise-induced weight loss on left ventricular diastolic function.. Am J Physiol Heart Circ Physiol.

[38] White TK, Wilson JE (1989). Isolation and characterization of the discrete N- and C-terminal halves of rat brain hexokinase: retention of full catalytic activity in the isolated C-terminal half.. Arch Biochem Biophys.

[39] Wilson JE (1995). Hexokinases.. Rev Physiol Biochem Pharmacol.

[40] Southworth R, Davey KA, Warley A, Garlick PB (2007). A reevaluation of the roles of hexokinase I and II in the heart.. Am J Physiol Heart Circ Physiol.

[41] Furuta H, Nishi S, Le Beau MM, Fernald AA, Yano H (1996). Sequence of human hexokinase III cDNA and assignment of the human hexokinase III gene (HK3) to chromosome band 5q35.2 by fluorescence in situ hybridization.. Genomics.

[42] Aleshin AE, Kirby C, Liu X, Bourenkov GP, Bartunik HD (2000). Crystal structures of mutant monomeric hexokinase I reveal multiple ADP binding sites and conformational changes relevant to allosteric regulation.. J Mol Biol.

[43] Aleshin AE, Zeng C, Bartunik HD, Fromm HJ, Honzatko RB (1998). Regulation of hexokinase I: crystal structure of recombinant human brain hexokinase complexed with glucose and phosphate.. J Mol Biol.

[44] Majewski N, Nogueira V, Bhaskar P, Coy PE, Skeen JE (2004). Hexokinase-mitochondria interaction mediated by Akt is required to inhibit apoptosis in the presence or absence of Bax and Bak.. Mol Cell.

