# Supplementary figures and images for: Regulation and Cytoprotective Role of Hexokinase III
Source: PLoS One. 2010 Nov 3;5(11):e13823. doi: 10.1371/journal.pone.0013823 (PMC2972215; doi:10.1371/journal.pone.0013823)

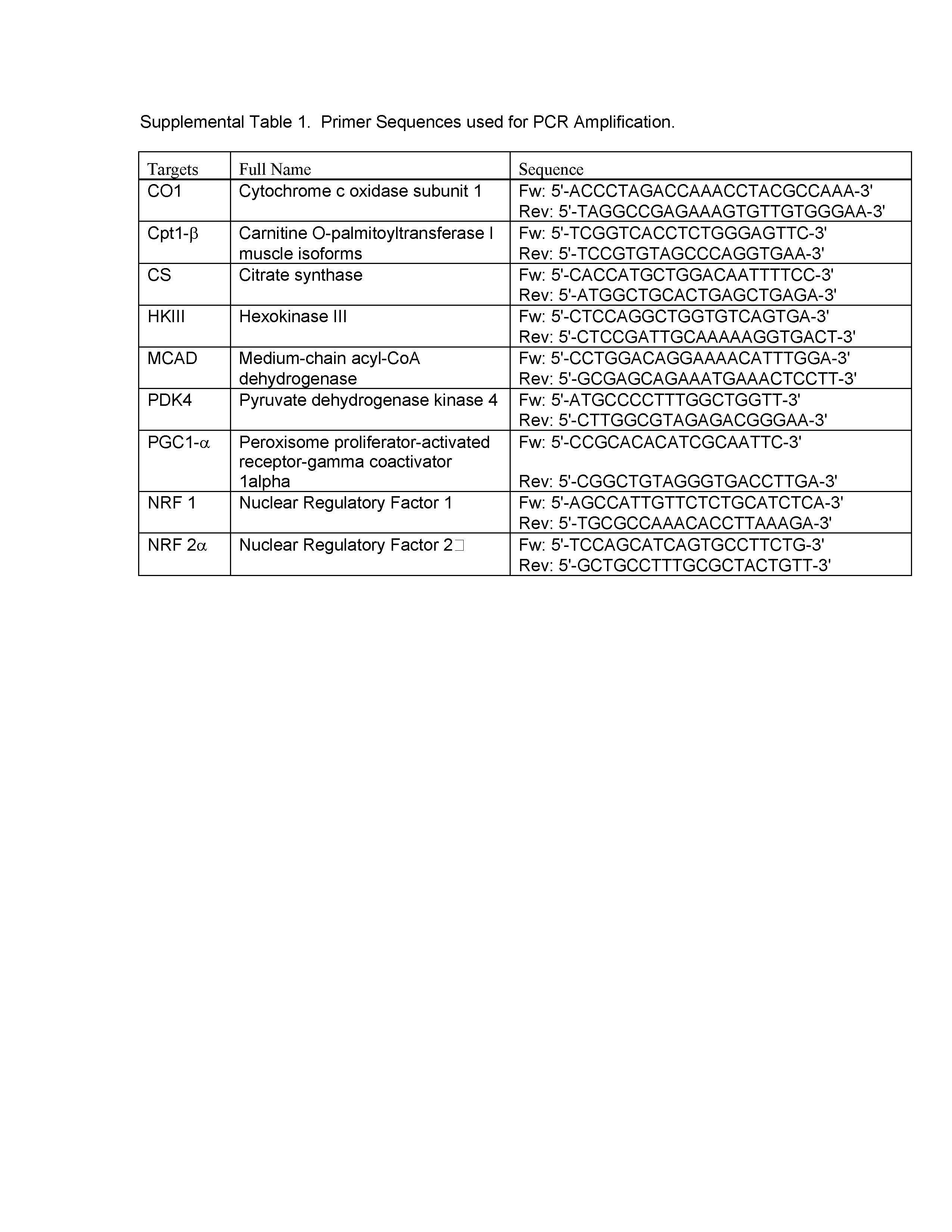

Supplement: Table S1 — Primer Sequences used for PCR Amplification (0.60 MB TIF) [file pone.0013823.s001.tif]

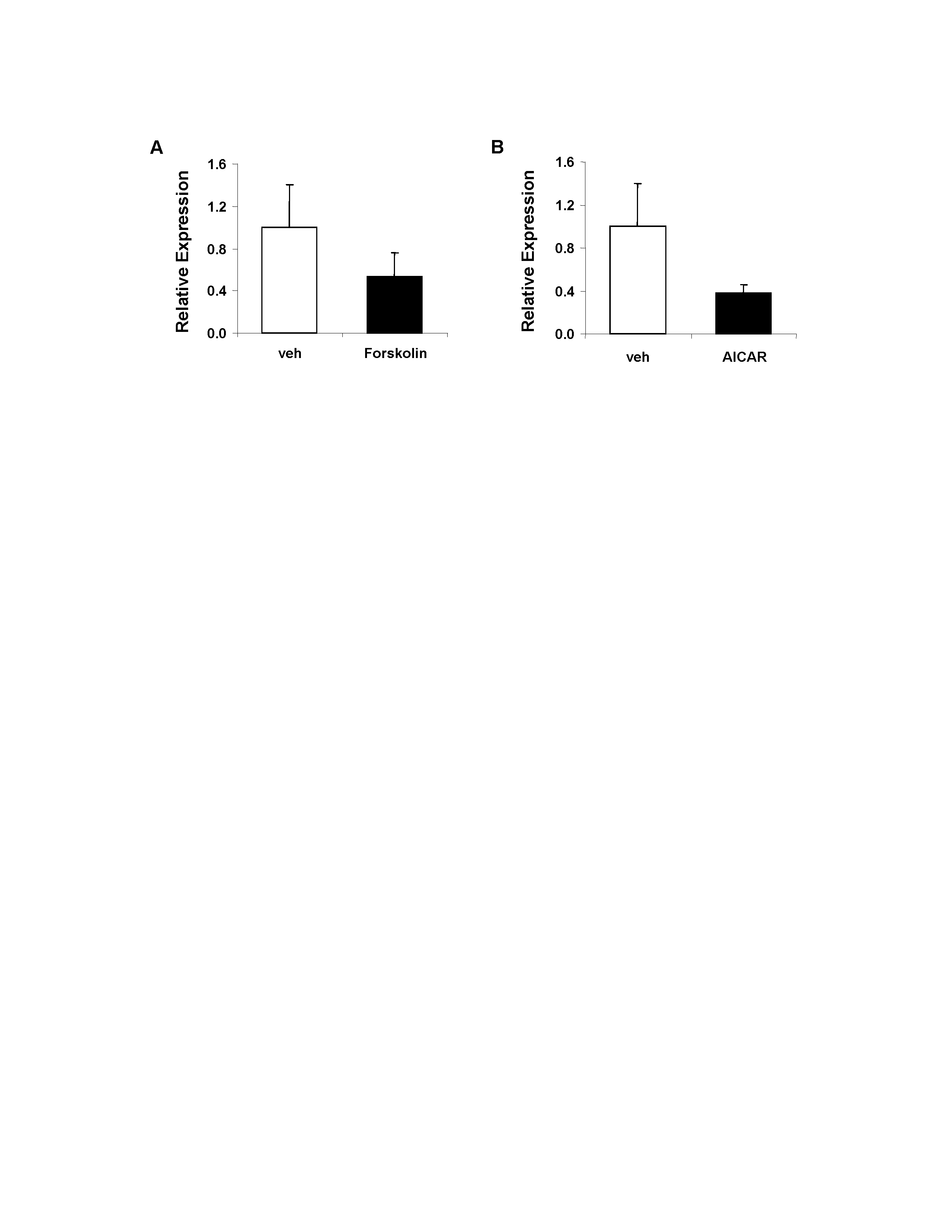

Supplement: Figure S1 — HKIII levels do not change in N1S1 cells after cAMP or AMPK activation. HKIII mRNA expression was measured in N1S1 cells cultured for 24 hours with or without (A) 5 microM forskolin (an inducer of cAMP production) or (B) 1 mM AICAR (an activator of AMPK). HKIII mRNA levels in treated and untreated cells were similar. Data are presented as mean+SEM, N = 3 for all groups. (0.52 MB TIF) [file pone.0013823.s002.tif]

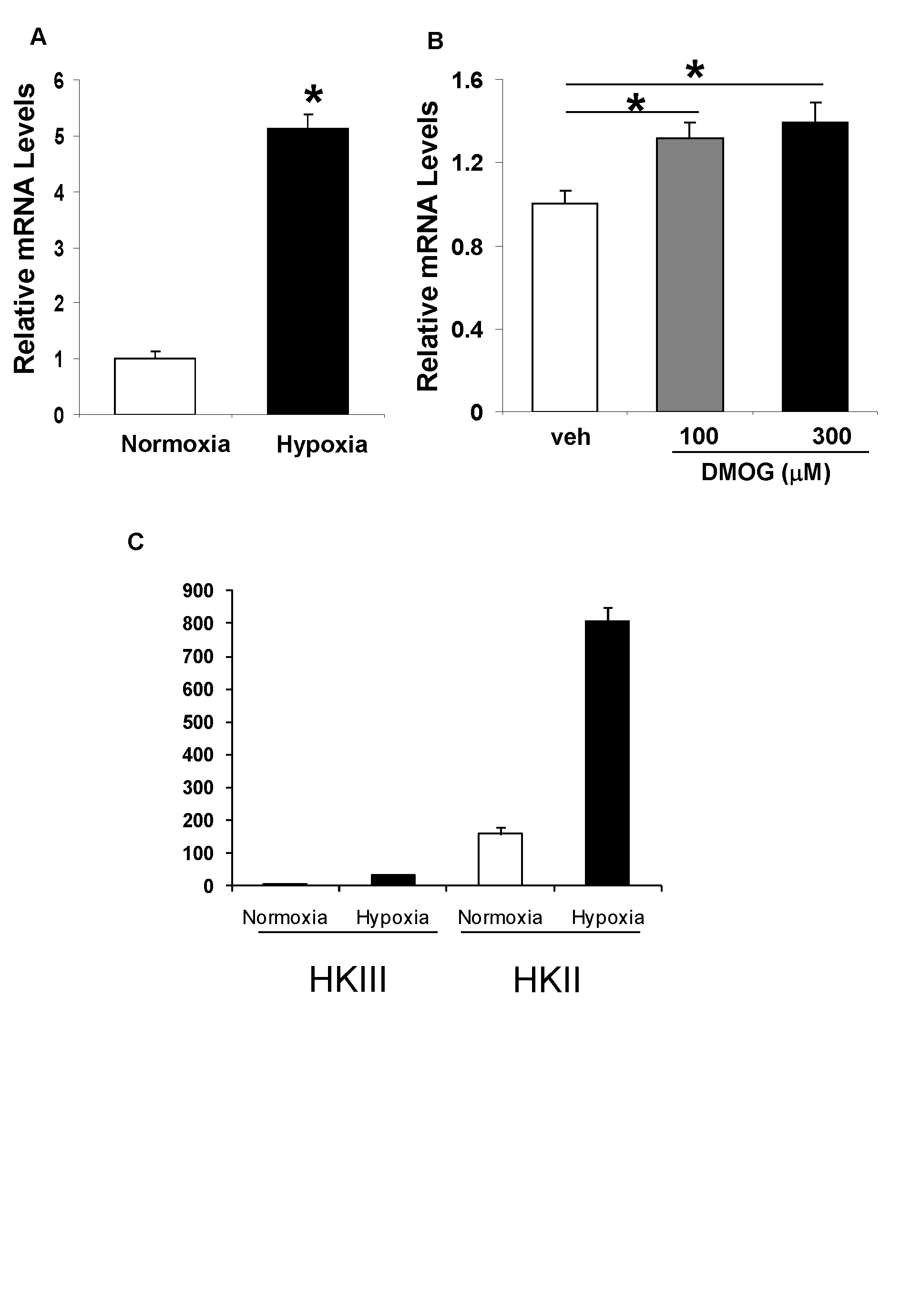

Supplement: Figure S2 — Hypoxia regulates HKII expression in N1S1 cells. (A) HKII mRNA levels were measured in N1S1 cells cultured under normoxic or hypoxic (1.5% O2) conditions for 24 hours. HKII levels were significantly increased after hypoxia treatment. (B) N1S1 cells were cultured under normoxic conditions for 24 hours with or without a HIF stabilizer (DMOG, 100 and 300 µM). HKII mRNA levels increased significantly after DMOG treatment. (C) Comparative mRNA expression levels of the HKII and HKIII isoforms in N1S1 cells cultured under normoxic or hypoxic (1.5% O2) conditions for 24 hours, demonstrating the higher levels of HKII expression in this cell type. *P<0.05 v. control; Data are presented as mean±SEM, N = 3–5 for all groups. (0.45 MB TIF) [file pone.0013823.s003.tif]

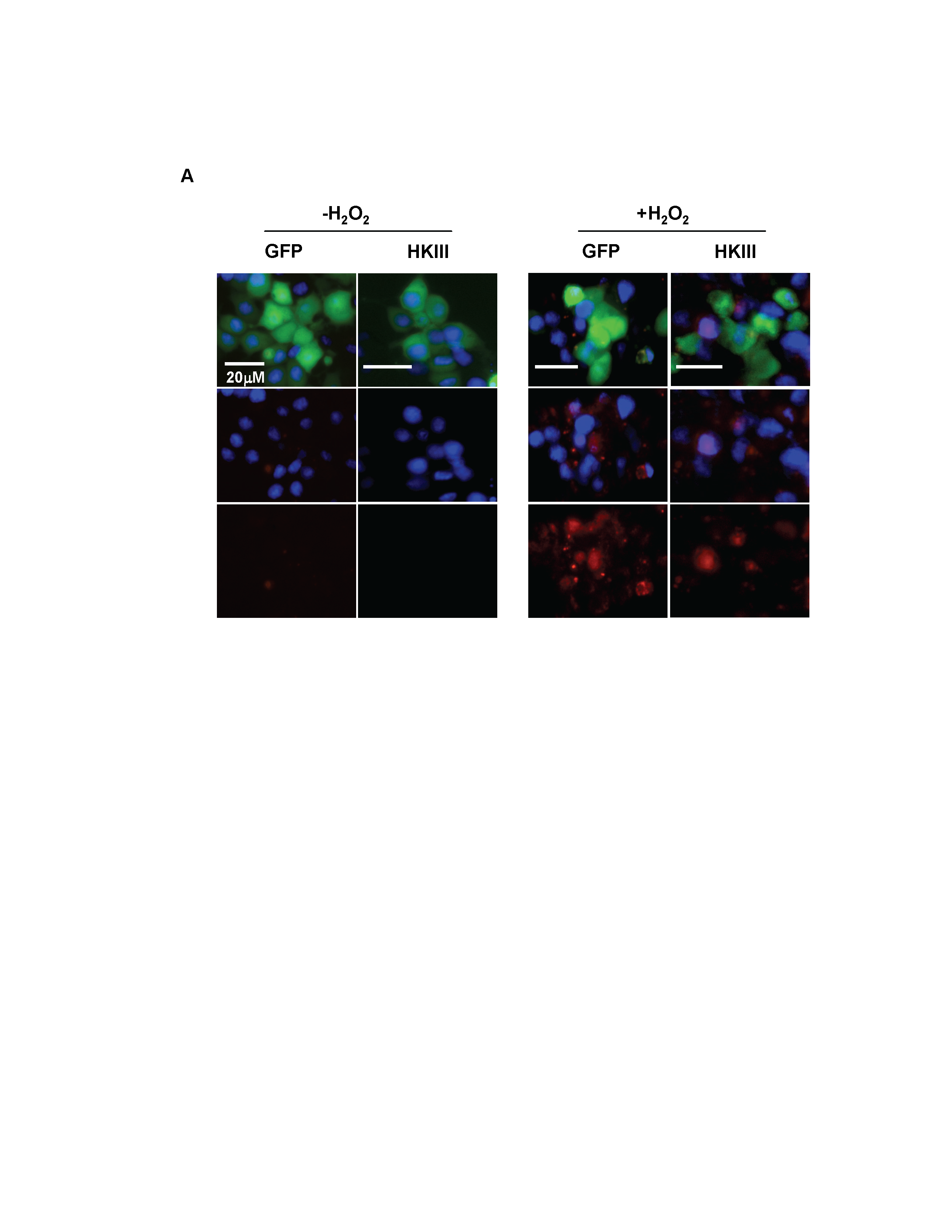

Supplement: Figure S3 — Overexpression of GFP and HKIII-GFP in HEK cells labeled with Mitosox. (A) GFP fluorescent overlay for the HEK cell images displayed in figure 5B, demonstrating the overexpression of the GFP and HKIII-GFP constructs in cells treated with or without 500 µM H2O2 (3 h), and then incubated with (MitoSox). Cell nuclei are labeled with Hoechst (blue). (2.61 MB TIF) [file pone.0013823.s004.tif]

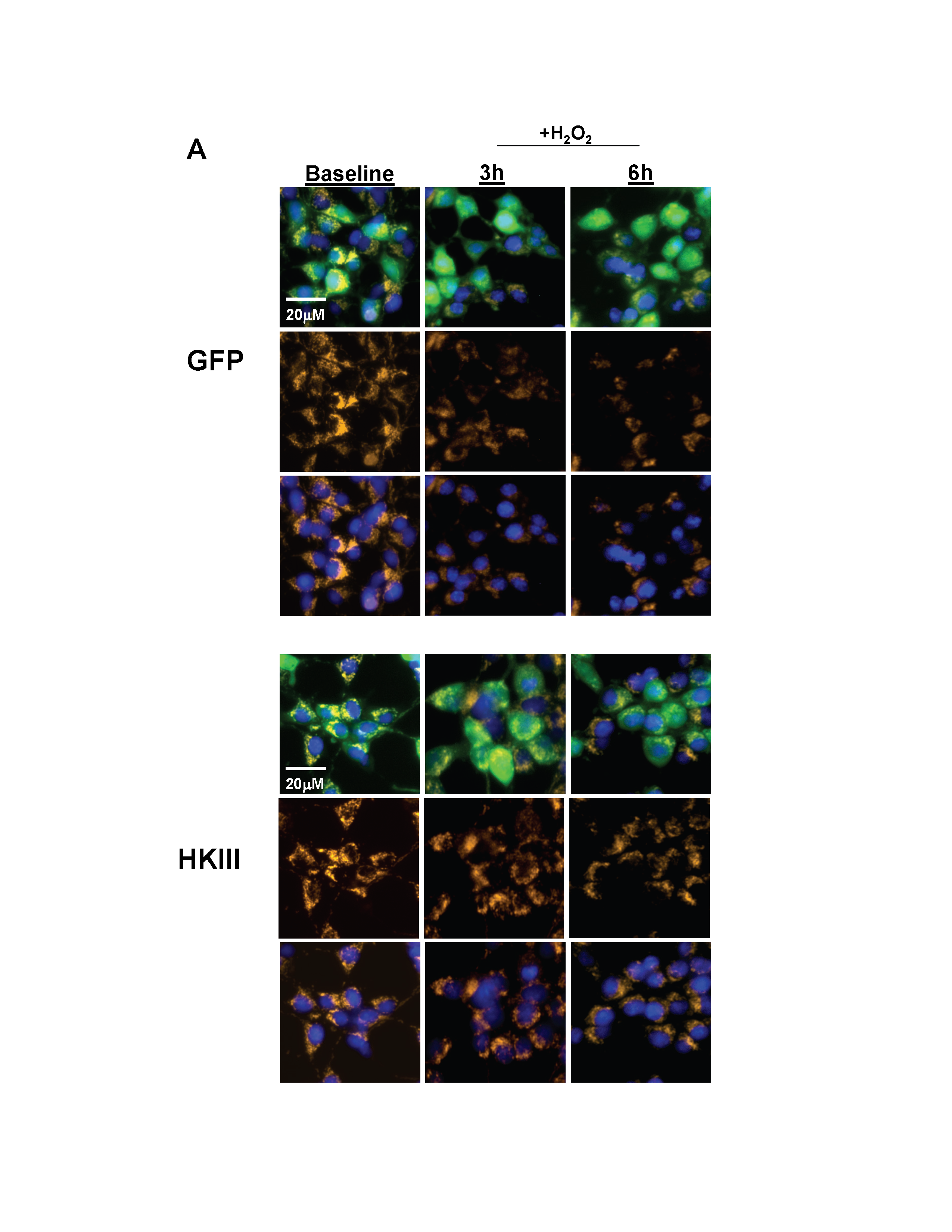

Supplement: Figure S4 — Overexpression of GFP and HKIII-GFP in HEK cells labeled with TMRE. (A) GFP fluorescent overlay for the HEK cell images displayed in figure 6 A-D, demonstrating the overexpression of the GFP (top panels) and HKIII-GFP (bottom panels) constructs in cells loaded with TMRE(orange) prior to the treatment condition indicated. Cell nuclei are labeled with Hoechst (blue). (5.19 MB TIF) [file pone.0013823.s005.tif]

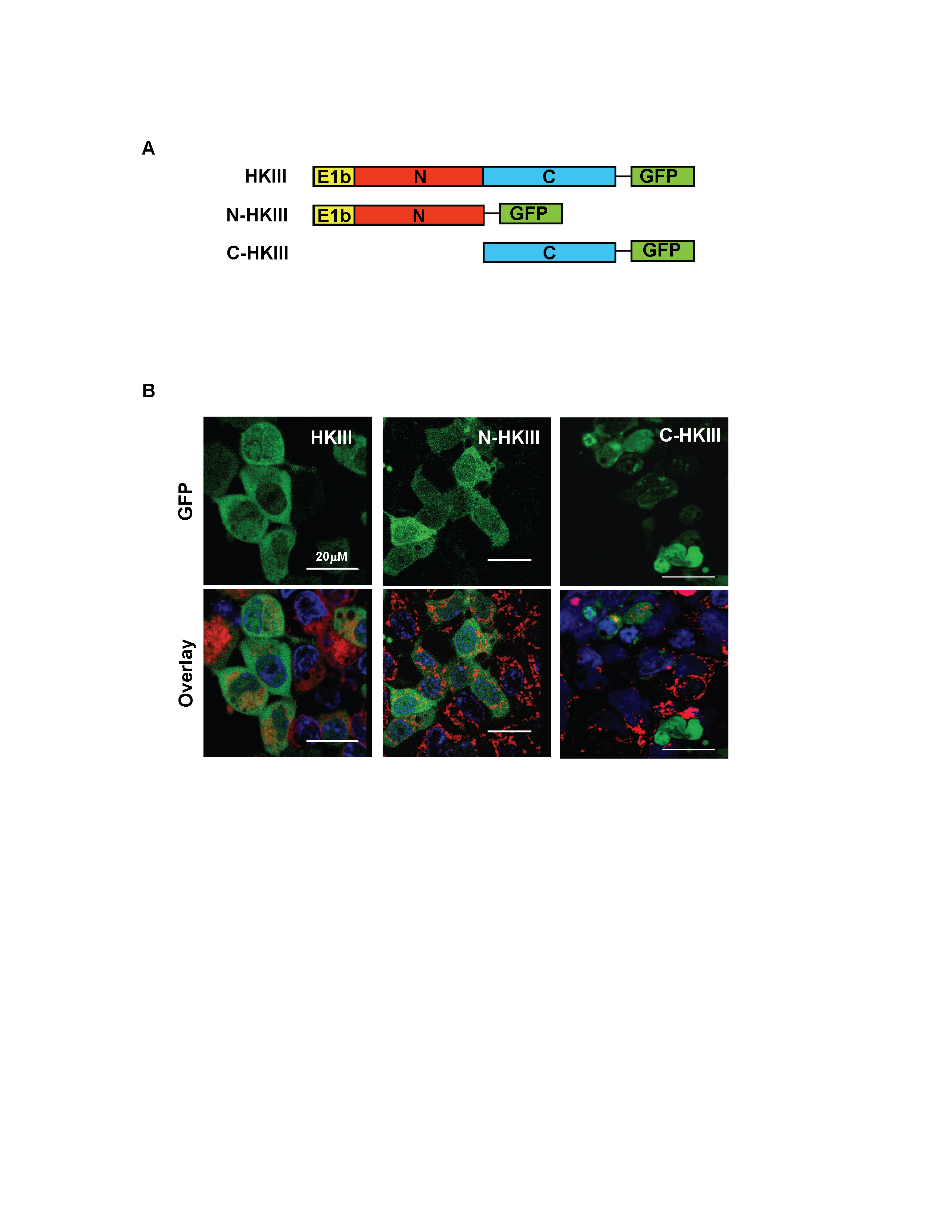

Supplement: Figure S5 — Overexpression of the N and C-terminal domains of HKIII in HEK293 cells. (A) Plasmid constructs coding for the expression of the N and C-terminal domains of HKIII. Each construct also coded for GFP expression at the C-terminus. (B) Live cell confocal images of HEK cells after transfection with the indicated constructs reveals soluble expression of the N-terminal half of HKIII (NHKIII) similar to the full length HKIII construct, while overexpression of the C-terminal domain resulted in aggregate formation. The cells were incubated with TMRE (red) to identify mitochondria, and nuclei were labeled with Hoechst (blue). (2.89 MB TIF) [file pone.0013823.s006.tif]

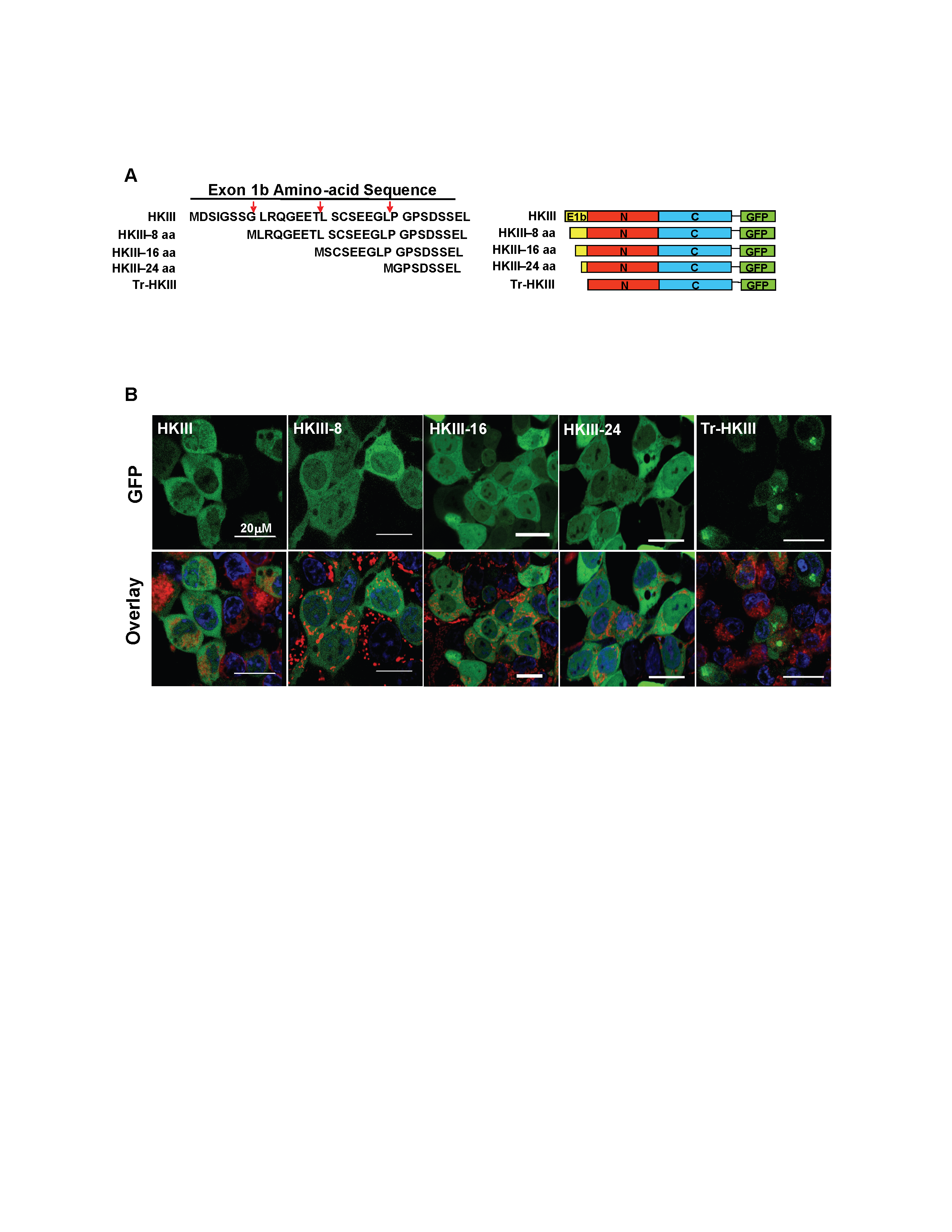

Supplement: Figure S6 — Progressive deletion of 8-amino-acid segments from the N-terminus of HKIII do not cause protein aggregation. (A) Plasmids expressing truncated versions of HKIII that lacked the first 8 (HKIII-8), 16 (HKIII-16), or 24 (HKIII-24) amino acids of the N-terminal sequence encoded by exon 1b were constructed and expressed in HEK293 cells; each construct also coded for GFP expression at the C-terminus. (B) Live cell confocal images of HEK cells after transfection with the indicated constructs showed that the full-length protein and each of the three serial truncation constructs were expressed in the cytoplasm and did not form cellular aggregates. The cells were incubated with TMRE (red) to identify mitochondria, and nuclei were label with Hoechst (blue). (3.35 MB TIF) [file pone.0013823.s007.tif]

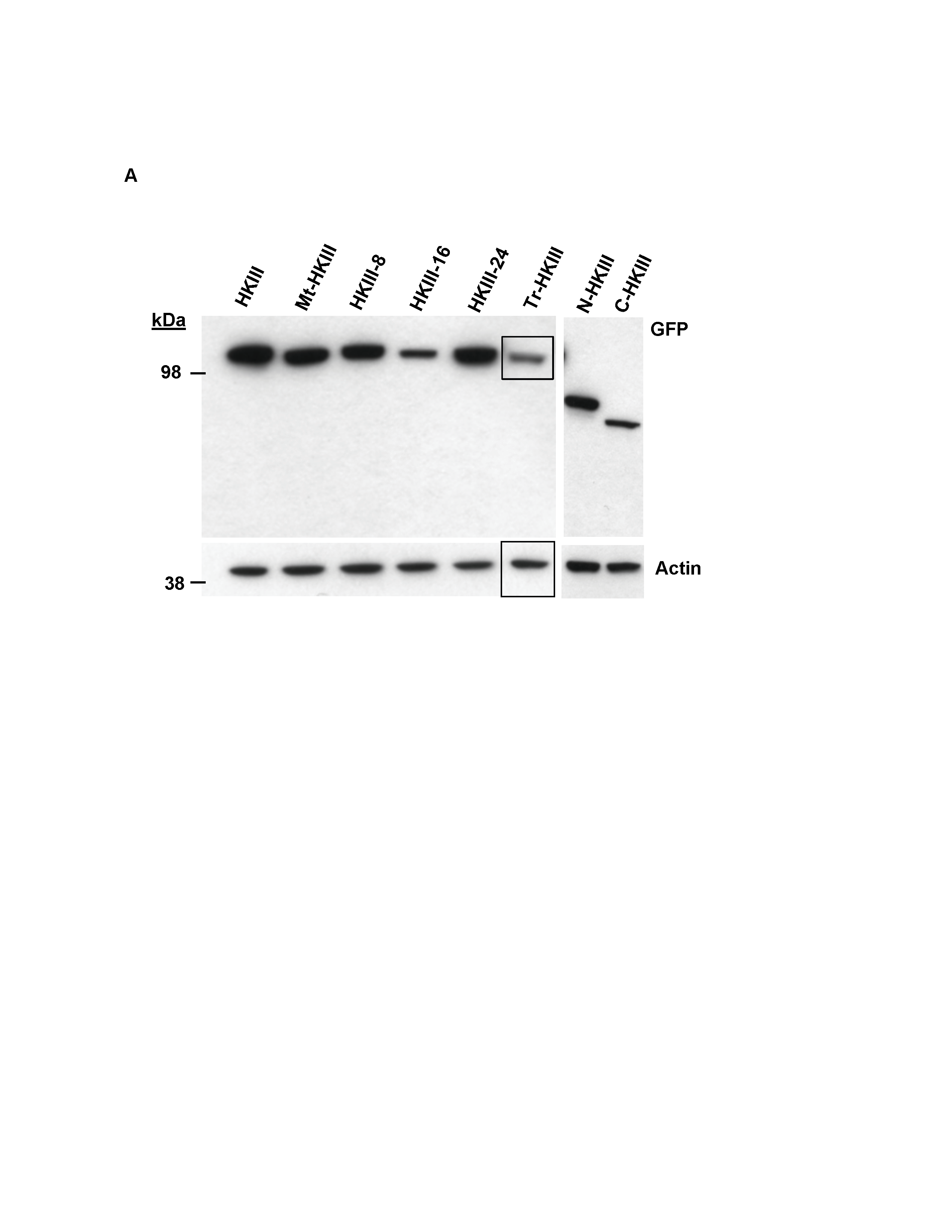

Supplement: Figure S7 — Western blot for protein expression for the HKIII constructs. (A) Western blot of cell lysates from HEK293 cells overexpressing the indicated constructs. For each construct, 1.5 µg of plasmid DNA was transfected into each well using Lipofectamine 2000 as described. (1.33 MB TIF) [file pone.0013823.s008.tif]
